# Application of Sapphire-Fiber-Bragg-Grating-Based Multi-Point Temperature Sensor in Boilers at a Commercial Power Plant

**DOI:** 10.3390/s19143211

**Published:** 2019-07-21

**Authors:** Shuo Yang, Daniel Homa, Hanna Heyl, Logan Theis, John Beach, Billy Dudding, Glen Acord, Dwyn Taylor, Gary Pickrell, Anbo Wang

**Affiliations:** 1 Center for Photonics Technology, The Bradley Department of Electrical and Computer Engineering, Virginia Polytechnic Institute and State University (Virginia Tech), Blacksburg, VA 24061, USA; 2 Department of Material Science and Engineering, Virginia Polytechnic Institute and State University (Virginia Tech), Blacksburg, VA 24061, USA; 3 Central Steam Plant, Facilities Department, Virginia Polytechnic Institute and State University (Virginia Tech), Blacksburg, VA 24061, USA

**Keywords:** single-crystal sapphire fiber, fiber Bragg gratings, distributed sensing, temperature sensing, wavelength multiplex, femtosecond laser, boiler

## Abstract

Readily available temperature sensing in boilers is necessary to improve efficiencies, minimize downtime, and reduce toxic emissions for a power plant. The current techniques are typically deployed as a single-point measurement and are primarily used for detection and prevention of catastrophic events due to the harsh environment. In this work, a multi-point temperature sensor based on wavelength-multiplexed sapphire fiber Bragg gratings (SFBGs) were fabricated via the point-by-point method with a femtosecond laser. The sensor was packaged and calibrated in the lab, including thermally equilibrating at 1200 °C, followed by a 110-h, 1000 °C stability test. After laboratory testing, the sensor system was deployed in both a commercial coal-fired and a gas-fired boiler for 42 days and 48 days, respectively. The performance of the sensor was consistent during the entire test duration, over the course of which it measured temperatures up to 950 °C (with some excursions over 1000 °C), showing the survivability of the sensor in a field environment. The sensor has a demonstrated measurement range from room temperature to 1200 °C, but the maximum temperature limit is expected to be up to 1900 °C, based on previous work with other sapphire based temperature sensors.

## 1. Introduction

A secure and efficient source of energy is critical to the stability of nations, as well as the health and welfare of citizens. To approach full utilization of power plants, there is a need to improve operating efficiencies, increase reliability to minimize downtime, and improve adherence to regulatory environmental policies [1,2]. Sensor and control technologies provide the foundation that will enable operators to fully utilize these technological advances for reliable system integration, addressing cybersecurity concerns, and optimizing plant operations. Techniques that utilize optical pyrometers, precious metal thermocouples, and acoustic pyrometers are used primarily for detection and prevention of catastrophic events and are typically deployed for single-point measurement [1,2,3,4]. Furthermore, these devices are limited by temperature and can be cost prohibitive or invasive if deployed in large numbers to obtain distributed temperature measurements [1,2,3,4]. Mature fiber optic sensing technologies are attractive options for these applications, due to their (partly) insusceptibility to electromagnetic interference (EMI). In addition, they offer distributed measurements over long distances with high accuracy and low noise [5]. Nonetheless, the extremely high temperatures and harsh environments have restricted the implementation in power plants. The most widely used optical fiber material, fused silica, is not able to withstand the chemically corrosive environments at temperatures in excess of 800 °C [6,7].

Single-crystal sapphire fibers are attractive for construction of sensors for various harsh environments owing to sapphire’s high melting point (~2040 °C), chemical stability, and optical transparency [8,9,10,11,12,13,14,15,16,17,18,19,20,21,22,23,24,25]. Distributed temperature sensing based on single-crystal sapphire fiber has been reported via Raman backscattering [12] or multiplexed fiber Bragg gratings (FBGs) [13,14,15,19]. Though the Raman backscattering-based sensing offers fully distributed measurements, the low Raman scattering cross-section of sapphire demands use of a high power laser, highly sensitive detection system, and a long exposure time, which raises the cost of the sensing system [12,16]. Nevertheless, FBGs have been demonstrated as effective sensing elements to achieve quasi-distributed sensing with flexible configurations, a fast response time, and low costs [17,18,19,20,21,22,23,24,25,26,27]. FBGs in single-crystal sapphire (SFBGs) have been demonstrated for sensing applications up to 1900 °C [22]. In this regard, the temperature response of SFBGs is twice as sensitive when compared to fused silica, owing to sapphire’s larger thermal expansion coefficient [22]. Although various SFBG-based sensing applications have been reported, to the best of our knowledge, this reported work primarily evaluates the sensing performance in a laboratory environment or under a controlled environmental conditions [11,13,14,15,19,20,21,22,23,25]. However, the deployment and evaluation of a sensor in the real field environment is crucial and offers valuable information for practical applications.

In this work, the deployment of a wavelength-multiplexed-SFBG-based multi-point temperature sensor in coal-fired and gas-fired boilers is reported. The sensor was fabricated, packaged, and calibrated in a laboratory environment. A 110-h isothermal test was conducted on the packaged sensor and the evolution of the spectral shape induced by spontaneous variation of intermodal coupling was observed and discussed. The procedure of the sensor deployment was introduced and its performance was evaluated. This work extends our preliminary results presented in previous work [24].

## 2. Materials and Methods

### 2.1. Fabrication and Working Principle of SFBGs Sensor

**Figure 1:**
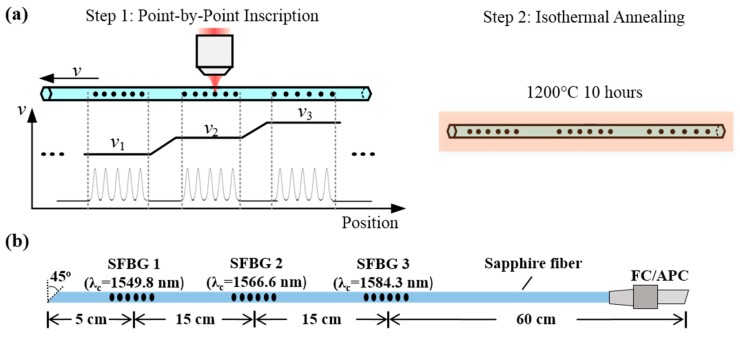
(**a**) Fabrication procedure for wavelength-multiplexed SFBGs via point-by-point method [15]. (**b**) Configuration of the fabricated sensing fiber. Note: SFBG = sapphire fiber Bragg grating.

Three SFBGs were inscribed in a 125 μm-diameter air-clad single-crystal sapphire fiber (MicroMaterial Inc., Tampa, FL, USA) via the point-by-point method with a femtosecond pulsed laser. The details of the fabrication procedure are described in previous work [15] and the grating configuration is shown in Figure 1b. The choice of the point-by-point method instead of the one via phasemask [11,14] is due to its excellent flexibility in fabricating cascaded SFBGs. When a broadband light is launched into a SFBG, only the light at or near the Bragg wavelength shows strong constructive interference and is efficiently reflected. The Bragg wavelength is defined as [18]:

(1) $$m\lambda_{B}=2n_{eff}\left(\varepsilon ,T\right)\Lambda \left(\varepsilon ,T\right),$$

where *m* is the order of the grating, *n_eff_* is the effective refractive index of the a given propagation mode, Λ is the grating pitch, *ɛ* is strain, and *T* is temperature. SFBGs with a desired Bragg wavelength can be easily achieved by tuning the grating pitch during fabrication. In this work, the center wavelengths of the fundamental mode in the gratings are 1549.8, 1566.6, and 1584.3 nm, respectively. Each grating was ~2 mm long and the inscription time was 2 s. Since the reflectivity of the gratings are weak (~0.6%) [15], the input and the far end of the sapphire fiber was polished to 7 degrees (Ferrule Connecter/Angled Physical Contact (FC/APC)) and 45 degrees, respectively, to minimize the influence from the interfacial Fresnel reflection. The inscribed FBGs were then annealed at 1200 °C for 10 h to enhance and stabilize their reflectivity [15].

**Figure 2:**
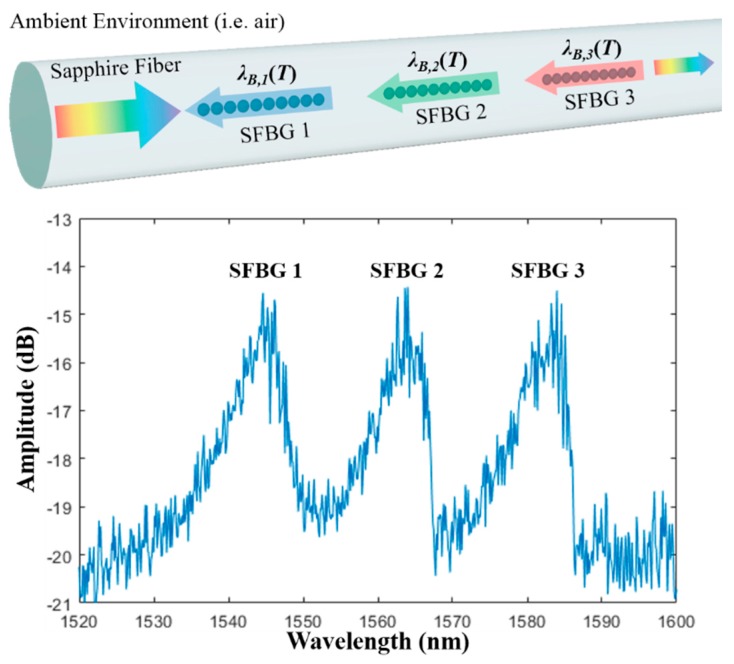
Working principle of wavelength-multiplexed-SFBGs temperature sensor and their spectra after fabrication at room temperature. Note: SFBG = sapphire fiber Bragg grating.

As indicated in Equation (1), both the effective refractive index and grating pitch are a function of strain and temperature. By implementing the sapphire fiber under a strain-free condition, the temperature information can be extracted by tracking the shift of the Bragg wavelength of an SFBG, Δ*λ_B_*, which is given by [24]:

(2) $$\Delta \lambda_{B}=\lambda_{B}\left(\alpha +\sigma \right)\Delta T,$$

where *α* and *σ* are the thermal expansion coefficient and thermo-optic coefficient, respectively. For sapphire fibers, *α* = 5.4 × 10^−6^/K and *σ*~1.2 × 10^−6^/K at 633 nm [24]. Since SFBGs at different locations have different Bragg wavelengths, temperature information at different locations can be extracted, as shown in Figure 2. Figure 2 also shows the spectra of the SFBGs after fabrication at room temperature. Since the ambient environment serves as the cladding layer, the large refractive index difference (~1.75 to 1) makes the waveguide highly multimode. As a result, the reflection spectrum of each SFBG is broadband (FWHM ~10 nm). To prevent spectral overlap, the Bragg wavelength of each SFBG was set at least 15 nm apart and the number of gratings was limited by the bandwidth of the light source. The spectral shape depends on the modal distribution seen by the grating, which is related to both the modal excitation of the launching fiber and the intermodal coupling induced by any perturbations during the light propagation [13].

### 2.2. Sensor Packaging

**Figure 3:**
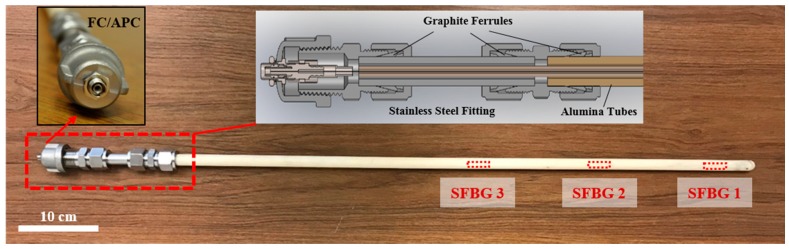
Design and picture of the sensor packaging.

The sensing fiber was packaged in a customized “tube-in-tube” design, as illustrated in Figure 3. The sapphire fiber was inserted in a high-purity alumina ceramic tube (Inner Diameter (ID): 1.57 mm, Outer Diameter (OD): 4.75 mm). There was no fixation of the fiber, which lay freely on the inner wall. This assembly was then inserted into another ceramic tube (ID: 6 mm, OD: 13 mm) with one sealed end. Customized stainless steel tube fittings were designed to secure the ceramic tubes, as shown in the inset of Figure 3. Given the rigidity of alumina and the potential thermal expansion mismatch, compressive fittings with soft graphite ferrules instead of epoxy were used to centralize and fix all tubes in place. A female standard FC/APC connector was installed at the end of the probe, eliminating the need for on-site splices and simplifying the installation process for the sensor probe. The details of all the components are listed in Table 1 and no special cleaning or care was performed for all the material before the packaging.

### 2.3. Interrogation System and Sensor Calibration

**Figure 4:**
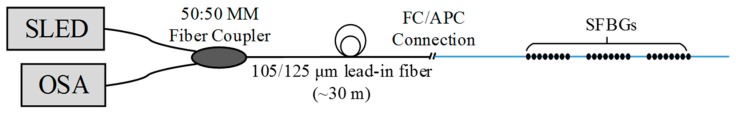
Scheme for the interrogation system. Note: SLED = superluminescent light emitting diode; OSA = optical spectrum analyzer; MM = multimode; FC/APC = Ferrule Connecter/Angled Physical Contact.

The packaged sensor was first calibrated and tested in a laboratory environment, as mentioned in Section 1. The interrogation system is illustrated in Figure 4. A superluminescent light emitting diode (SLED) with a center wavelength of 1565 nm and bandwidth of 80 nm (Model: S5FC1005P, Thorlabs Inc., Newton, NJ, USA) was used as the illumination source. An approximately 30-m long commercial step-index 105/125 μm multimode silica fiber was chosen as the lead-in fiber due to its capability to excite a sufficient amount of modes in the sapphire fiber and high coupling efficiency of the reflected light. A 50:50 multimode coupler of the same type of fiber was used to launch and collect the light. An optical spectrum analyzer (OSA) (Model: OSA 203, Thorlabs Inc., Newton, NJ, USA) was used to record the reflection spectrum with a resolution and accuracy of 140 pm and ±4 pm, respectively. The sensors were inserted into a tube furnace (Model: GSL 1500X, Richmond, CA, USA) for calibration. Since the high modal volume of the air-clad sapphire fiber broadens the reflection peak, an empirical curve fitting with a double-Gaussian model was applied to determine the average Bragg wavelength [15]. After the calibration, the durability of the packaged sensors was evaluated by being exposed to a temperature of 1000 °C for 110 h and the spectrum was acquired every 15 min without averaging. The choice of this temperature is based on the estimated working temperature of a commercial boiler.

## 3. Results

### 3.1. Sensor Calibration and Stability

**Figure 5:**
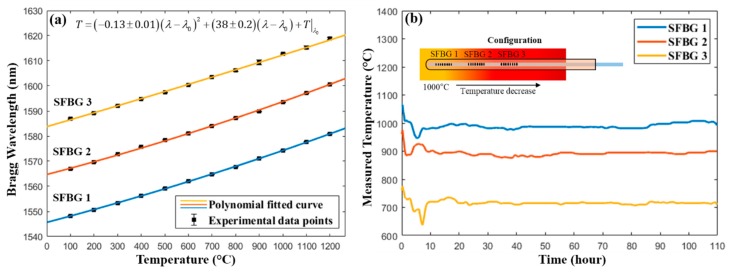
(**a**) Calibration of the temperature response of the SFBGs, where *λ* (nm) is the measured wavelength, *λ*_0_ (nm) is the Bragg wavelength at room temperature, and *T* (°C) represents the temperature. (**b**) Evolution of the demodulated temperature during the 110-h isothermal test. The insert shows the configuration of the FBGs during the test. Note: SFBG = sapphire fiber Bragg grating. FBG = fiber Bragg grating.

The sensor was calibrated from room temperature to 1200 °C and the response was evaluated by a second-order polynomial fitting, as shown in Figure 5a. All three gratings exhibited a highly linear response with a fitted coefficient ~38 °C/nm (or ~26.3 pm/°C). In the package durability test, the SFBGs were put under a thermal gradient (see Figure 5b), where SFBG 1 and SFBG 3 were at the hottest and coldest locations, respectively. The choice of this configuration is so as to mimic the actual test configuration, as described in Section 3.2. The demodulated temperature during the 110-h test, as shown in Figure 5b, has a strong fluctuation in the first 10 h, but stabilizes after that. The cause of such fluctuation will be explained in the following. The mean and standard deviation of the measured temperature after the first 10 h were: SFBG 1: *T*_mean_ = 989.6 °C, *σ* = 7.35 °C, SFBG 2: *T*_mean_ = 890.7 °C, *σ* = 5.26 °C, SFBG 3: *T*_mean_ = 715.9 °C, *σ* = 4.27 °C. There is a temperature difference between the maximum furnace temperature (1000 °C) and the SFBG 1 (989.6 °C), due to a temperature offset which is caused by a discrepancy between the position of the thermocouple and the SFBGs sensor. The distributed measurement capability of the SFBG sensor array was also verified by the characterization of the thermal gradient in the furnace.

**Figure 6:**
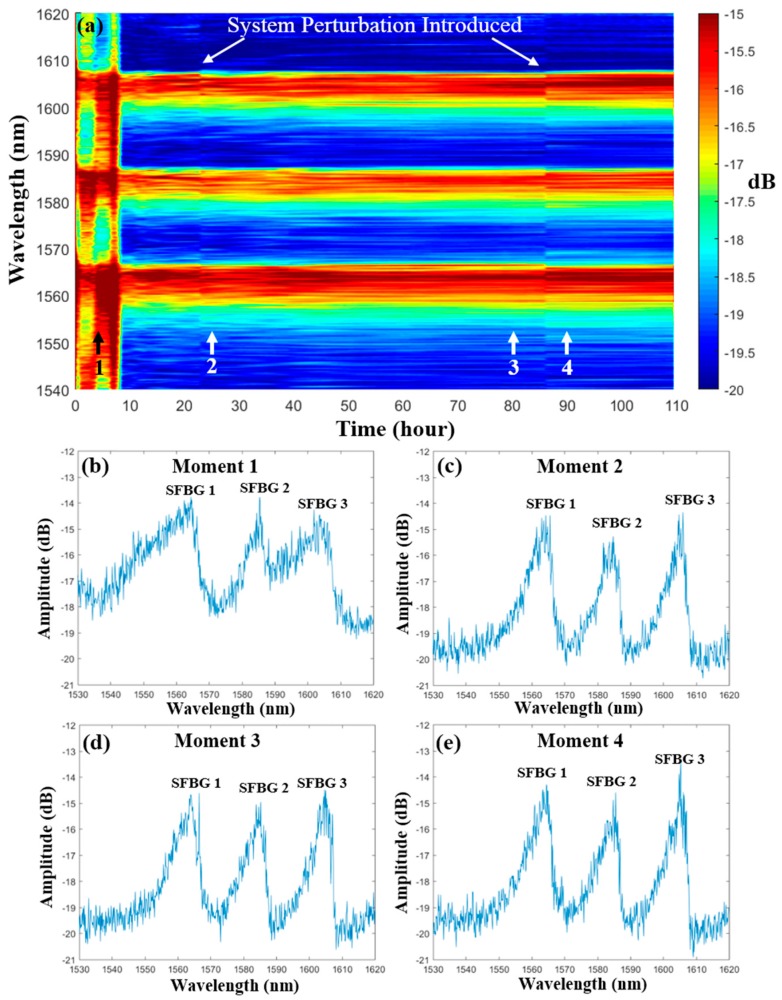
(**a**) Evolution of the reflection spectrum during the 110 h test. The spectra at selected moments are shown (**b**–**e**).

The evolution of the reflection spectrum after 110 h is shown in Figure 6a, in which all of the spectra are normalized with respect to the SLED source. The spectrum was unstable during the first 10 h, which is caused by the contamination during the packaging process [13]. In this regard, these deposited contaminants at the sapphire fiber surface start to decompose or vaporize at elevated temperature. During this process, the morphology of the fiber surface constantly changes. As indicated in Section 2.1, the spectral shape can be altered by intermodal coupling induced by any perturbations during the light propagation. Thus, random variation of the reflection spectral shape was observed, as shown in Figure 6a,b. After prolonged dwelling time, the contaminants burn off completely and the core-clad interface returns to a pristine state, which explains the disappearance of the dramatic changes in the spectral shape after the first 10 h. Furthermore, it explains the fluctuation of the demodulated temperature during the first 10 h, since the peak fitting requires a relatively stable spectral shape. This result indicates that a protective layer or packaging may be needed for air-clad SFBG sensors in practical applications. In addition, the probe was annealed at 1000 °C for 12 h before the actual deployment to stabilize the spectrum.

After the first 10 h, the evolution of the spectrum continues due to the intermodal coupling induced by the fluctuation of the ambient environment, such as temperature and vibration. In addition, perturbation to the system was deliberately introduced at 23 h and 86 h by repositioning the leading fibers or rotating the probe. Sudden changes of the spectral shape were observed (vertical lines in Figure 6a), which further confirms that the spectral shape is highly sensitive to the modal excitation and intercoupling. By comparing the spectra at different moments (see Figure 6c–e), the intermodal coupling induced by either ambient variation (Moment 2 and 3) or manual perturbation (Moment 3 and 4) mainly affects the ripples on a reflection peak. However, the overall spectral shape is relatively stable. Hence, peak fitting remains a simple and effective approach to characterize the reflection spectrum of a SFBG. The standard deviation values of the Bragg wavelength demodulated with the peak fitting method after the first 10 h were 0.19 nm, 0.14 nm, and 0.11 nm for SFBG 1, SFBG 2, and SFBG 3, respectively.

### 3.2. Sensor Deployment and Performace

#### 3.2.1. Coal-Fired Boiler

**Figure 7:**
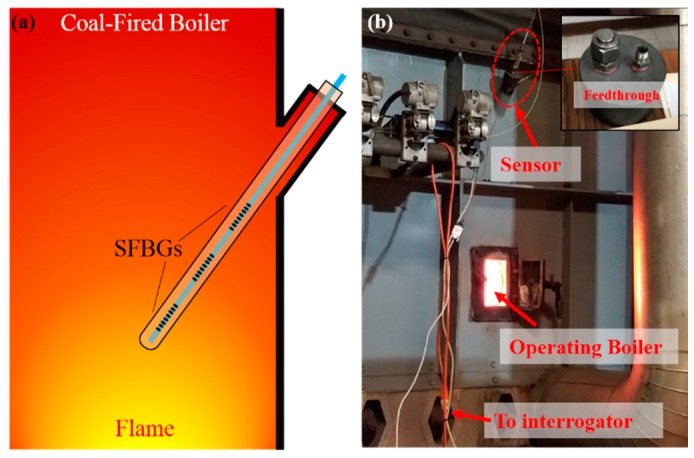
Sensor deployment in a coal-fired boiler: (**a**) scheme for the configuration of the sensor (thermocouple not shown); (**b**) picture of the deployment site.

The packaged sensor was installed in a commercial coal-fired boiler, with the accompanying interrogation system installed nearby, as described in Section 3.2.3, in the Virginia Tech Central Steam Plant, which is a commercial facility that provides electric service to the surrounding community. A commercially available K-type thermocouple probe sheathed with nickel-chrome-based material (Model: super OMEGACLAD™ series, OMEGA Engineering Inc., Norwalk, CT, USA) was packaged as the same design as the sensor probe and installed to provide a temperature reference. Due to space limitations, only one thermocouple probe was installed near the location of SFBG 1. A pre-existing inlet hole cap on the boiler was modified to include two ports with welded stainless steel compression fittings for the fiber-optic sensor and a thermocouple (the insert of Figure 7b). As shown in Figure 7a, the sensor was installed and positioned nearest to the boiler’s hot zone for maximum temperature exposure. SFBG 1 is closer to the flame and SFBG 3 is located nearest to the boiler wall. To prevent potential thermal shock, the sensor probe was slowly inserted into the boiler and the response time of the sensor was estimated to be less than 10 s during the insertion. Moreover, the installation of the sensors and the thermocouple did not require a shutdown of the boiler or special accommodation. The sensor deployment was performed seamlessly over a period of approximately 2 h. The measurement results were recorded every 5 min and monitored remotely in real time through a wireless internet connection.

**Figure 8:**
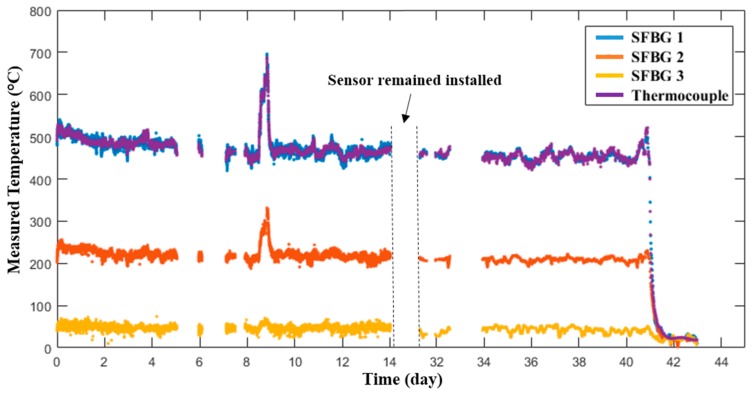
Temperature response of the sensor in a commercial coal-fired boiler over 42 days.

The sensing system operated for over 42 days and experienced a peak temperature of ~700 °C. In general, the temperature of the boiler was determined by the actual demands for power in the local area. Two distinct events were successfully detected during the test. The first one is a surge of temperature around Day 9, due to unexpected cold weather in the local region. The second one is a decrease of the temperature around Day 41, due to the routine maintenance of the boiler. The temperature gradient within the boiler can be readily seen by the temperature measurements provided by the three SFBGs, as shown in Figure 8. Furthermore, the prototype sensor measurements were consistent with those provided by the thermocouple co-located with SFBG 1. By taking the thermocouple as the reference, the measurement error during the entire test period was within ±20 °C and the standard deviation was 8.01 °C. The loss of temperature readings during the test was due to loss of power to the light source and was easily remedied by returning power to the unit. The loss of readings for ~14 days starting from Day 15 was due to damage to the interrogation system and components that appeared to be due to the local environment and incorrect handling of the operation team. However, the sensor probe remained installed during the entire test period. Unfortunately, the sensor probe broke at the end of the test due to improper handling. Thus, its performance after the retrieval was not evaluated.

#### 3.2.2. Gas-Fired Boiler

**Figure 9:**
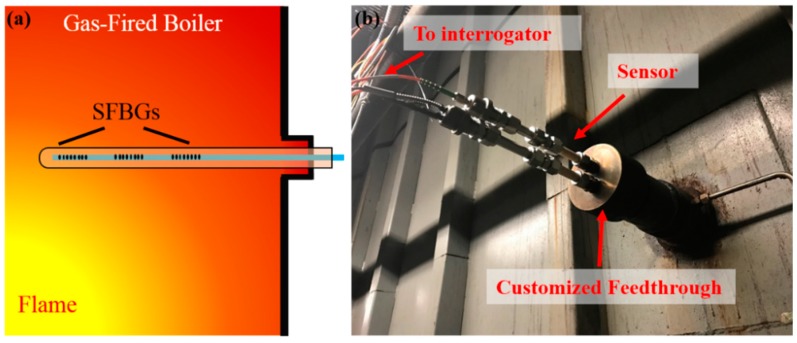
Sensor deployment in a gas-fired boiler: (**a**) scheme for the configuration of the sensor (thermocouple not shown); (**b**) picture of the deployment site.

The packaged sensor was installed in a commercial gas-fired boiler, with the accompanying interrogation system installed nearby, in the Virginia Tech Central Steam Plant with similar configuration and procedures to the one in coal-fired boiler (Figure 9). The probe was mounted horizontally instead of an oblique mount as in the coal-fired boiler, and the measurement results were also recorded every 5 min.

**Figure 10:**
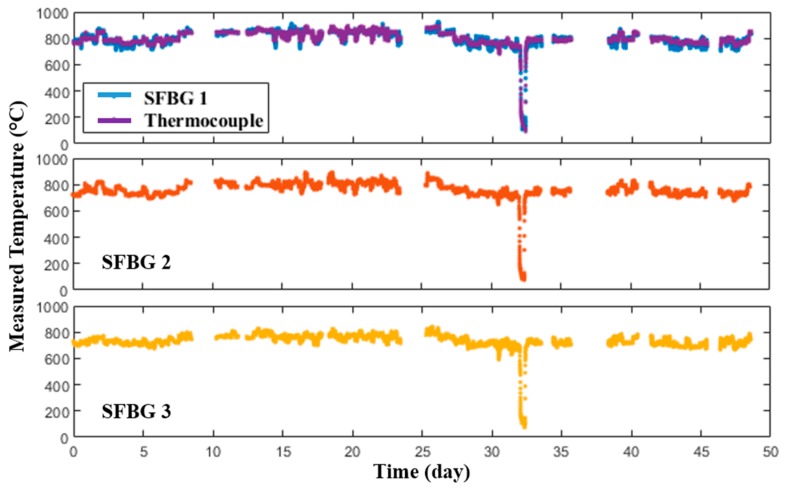
Temperature response of the sensor in a commercial gas-fired boiler over 48 days.

**Figure 11:**
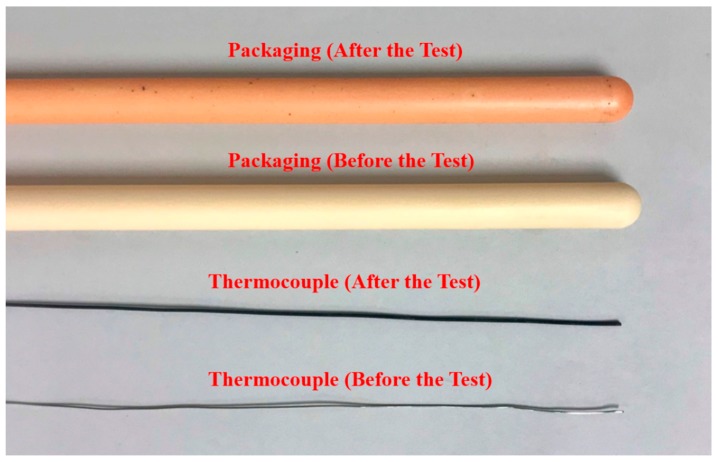
Picture of the physical appearances of the packaging and thermocouple before and after the test.

The sensing system operated for over 48 days and experienced a peak temperature of ~950 °C. A sudden drop of temperature around Day 33 was detected during the test, which was caused by a power outage induced by a thunderstorm in the local area. As shown in Figure 10, the temperature gradient within the boiler was less than the one in the coal-fired boiler, which was probably due to the horizontal positioning. Likewise, the prototype sensor measurements were consistent with those provided by the thermocouple co-located with the SFBG 1. By using the thermocouple as the reference, the measurement error during the entire test period was within ±40 °C and the standard deviation was 14.39 °C. There were also several data logging issues during the test caused by software and hardware issues in the interrogation system. However, the sensor probe remained installed during the entire test period. The sensor was successfully retrieved after the deployment and fully functional. Although there was a high strain at the mounting point due to the horizontal positioning, no observable deformation or crack was observed in the retrieved probe. The physical appearances of the packaging and thermocouple before and after the test are shown in Figure 11. There was a red layer near the end of the outer ceramic tubing where the probe experienced the highest temperature. Although the chemical composition and formation mechanism of this layer is still under investigation, this result indicates that proper packaging may be needed for the sensor deployed inside boilers. The thermocouple, though it is rated up to 1335 °C and was packaged with the same design as the sensor, turned black and rough after the test, showing that general commercial thermocouples are not an ideal choice for long-term application inside boilers. No change of appearance was observed on either the inner ceramic tubing or the sapphire fiber.

#### 3.2.3. Interrogation System and User Interface

**Figure 12:**
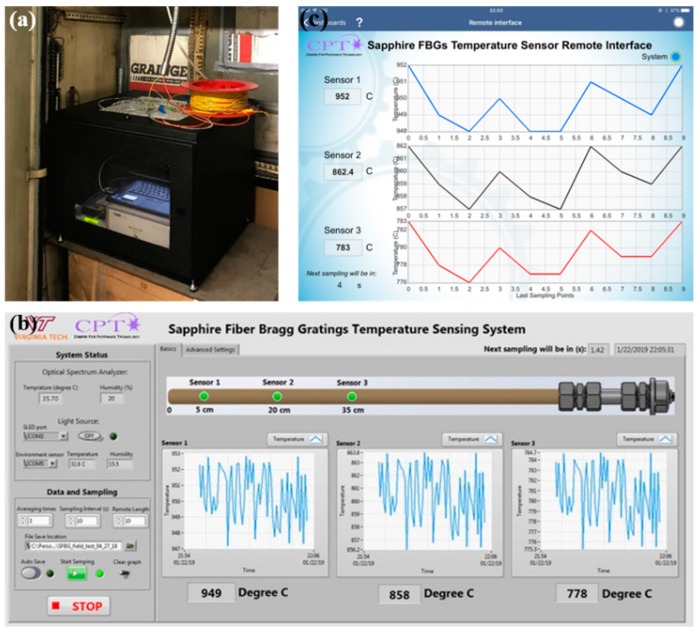
(**a**) Picture of the onsite interrogation system packaged in a weather-proof enclosure. (**b**) Onsite user interface. (**c**) Remote user interface.

The onsite interrogation system used in both the coal-fired and gas-fired boiler tests consists of all the parts listed in Section 2.3. Moreover, a weather-proof enclosure was used to protect the electronics from the field environment, as can be seen in Figure 12a. An onsite, user-friendly interface was developed via LabVIEW^TM^ for use by sensor engineers and end-users (operators). As shown in Figure 12b, real-time diagnostics can be performed for the temperature probe. Relevant information, such as the raw and the filtered sensor spectral responses. as well as the peak fitting parameters, are readily accessible for tuning and trouble-shooting. Remote access to data is often desired in daily applications due to the flexibility. Driven by this need, a read-only remote interface was developed, as shown in Figure 12c, via the LabVIEW Data Dashboard^TM^ App on iOS10^TM^ platform, allowing the user to visualize the data from anywhere through an internet connection.

## 4. Conclusions

In this paper, a multi-point temperature sensor based on three wavelength-multiplexed SFBGs was fabricated and packaged with a customized design. The sensor was calibrated in a laboratory environment and it showed great linearity of temperature response from room temperature to 1200 °C. During the 110-h isothermal test at 1000 °C, strong fluctuations of the spectral shape were observed within the first 10 h test, which most likely originated from the contamination during the packaging process. This result indicates that proper packaging or a protective layer may be needed for air-clad SFBG sensors in practical applications. Besides the dramatic change of the spectrum in the first 10 h, there was a constant spectral variation due to the intermodal coupling induced by ambient perturbations. However, those variations mainly affect the ripples on a reflection peak rather than its overall shape. Hence, the peak fitting method is an effective method to deduce the Bragg wavelength in practical applications and it can achieve <0.2 nm demodulation accuracy. In order to further improve the demodulation accuracy, the most straightforward method is to narrow the reflection peak. One strategy is to only excite fundamental or low-order modes in the sapphire fiber [20]. However, this method limits the length of the sapphire fiber section in front of the SFBG (<200 mm) and its stability in a field environment still needs to be investigated [13]. Another strategy is to use a smaller diameter sapphire fiber to reduce the modal density. For an air-clad sapphire FBG, the reflection peaks of different modes can be easily distinguished once the diameter is smaller than 20 μm and each peak has a <1 nm bandwidth [25]. The fabrication of a small diameter sapphire fiber can be achieved by laser-heated pedestal growth (LHPG) fabrication [28], in which diameters down to about 20 microns can be achieved, or by chemical-etching processing [29], in which diameters down to one micron or below can be achieved.

In summary, the packaged sensor was successfully deployed in a coal-fired and a gas-fired boiler in a commercial power plant. Installation of the sensors did not require a shutdown of the boiler or special accommodation. The sensor detected several unusual temperature fluctuations caused by local weather conditions, showcasing its effectiveness to monitor boiler temperatures within the power plant environment. The number of measurement points in this work was limited by the bandwidth of the light source. However, owing to the wide transparency window of sapphire (0.4–4 μm) [30], the number of measurement points can be easily increased by using a broader bandwidth source. This potential capability of distributed measurements with a single fiber offers an efficient way to deploy 2D or 3D thermal mapping compared to traditional techniques with thermocouples or pyrometers. In addition, the constant performance over the entire test period shows the sensor’s survivability in a field environment. Overall, it has been demonstrated that SFBG-based sensors are an excellent candidate for temperature measurements or mapping in commercial boilers.

## Figures and Tables

**Table 1:** Description of the Packaging Components.

Component	Material	Vendor
Ceramic Tube	High Purity Alumina (99.8%)	McDanel Advanced Ceremic, CoorsTek
Stainless Steel Fitting	304 Stainless Steel	McMaster Carr
Ferrule	Graphite	Ohio Valley Specialty
Fiber Connecter	Zirconia, Stainless steel	Thorlabs
